# EFFECTS OF IMPLEMENTING AGETECH TO SUPPORT DEMENTIA CARE IN A RURAL AND NORTHERN SETTING

**DOI:** 10.1093/geroni/igad104.1982

**Published:** 2023-12-21

**Authors:** Shannon Freeman, Sarah Sousa, Davina Banner-Lukaris, Kelly Skinner

**Affiliations:** University of Northern British Columbia, Prince George, British Columbia, Canada; University of Waterloo, Waterloo, Ontario, Canada; University of Northern British Columbia, Prince George, British Columbia, Canada; University of Waterloo, Waterloo, Ontario, Canada

## Abstract

Older adults wishing to age in northern and rural communities deserve equitable access to technologies that support optimal health, well-being, and quality of life. A new dementia care home opened in a rural and northern community in 2022 with consciously embedded multiple AgeTech solutions to enhance residents quality of life and care. The AgeTech solutions including hydroponic gardening, circadian lighting, and virtual exercise programming, were implementated into the 10-bed facility through a partnership between the Center for Technology Adoption for Aging in the North (CTAAN), health systems leaders, and community partners. The purposeful implementation of AgeTech within the facility through this partnership aimed to enhance the life-skills and living model of care to maximize independence, function, and resident well-being. We conducted a process evaluation assessing data from interviews with facility staff, health systems leaders, and secondary analysis of existing documentation to objectively measure the implementation process and inform a framework for ongoing assessment of AgeTech. Results highlight areas for ongoing technology innovation and inform policy decisions for future initiatives. Informants described the benefits in use of non-invasive circadian lighting technology to support resident well-being by enhancing sleep, rest/activity, reducing behaviours associated with dementia. Technology enhanced environmental well-being and expanded opportunities for residents to engage in meaningful activities. Participants also described the physical and social benefits for residents using individual and social multiplayer modes of exercise technologies. Providing equitable access to services across dispersed and sparse populations is challenging, yet with implementation of AgeTech quality of care can be enhanced.

